# Interleukin-1 beta promotes neuronal differentiation through the Wnt5a/RhoA/JNK pathway in cortical neural precursor cells

**DOI:** 10.1186/s13041-018-0383-6

**Published:** 2018-07-04

**Authors:** Shin-Young Park, Min-Jeong Kang, Joong-Soo Han

**Affiliations:** Biomedical Research Institute and Department of Biochemistry and Molecular Biology, College of Medicine, Hanyang University, 222 Wangsimni-ro, Seongdong-gu, Seoul 04763 Republic of Korea

**Keywords:** Interleukin-1 beta (IL-1β), Neuronal differentiation, Wnt5a, RhoA, C-jun N-terminal kinase (JNK)

## Abstract

Pro-inflammatory cytokine interleukin-1 beta (IL-1β) is a key mediator of inflammation and stress in the central nervous system (CNS), and is highly expressed in the developing brain. In this study, we investigated the possible role of IL-1β in neuronal differentiation of cortical neural precursor cells (NPCs). We showed that stimulation with IL-1β increased expression levels of neurotrophin-3 (NT3) and neurogenin 1 (Ngn1) and promoted neurite outgrowth. We also found that IL-1β increased mRNA and protein levels of Wnt5a. Knockdown of Wnt5a by transfection with Wnt5a siRNA inhibited IL-1β-induced neuronal differentiation. Moreover, IL-1β-induced Wnt5a expression was regulated by nuclear factor kappa B (NF-κB) activation, which is involved in IL-1β-mediated neuronal differentiation. To examine the role of Wnt5a in neuronal differentiation of NPCs, we exogenously added Wnt5a. We found that exogenous Wnt5a promotes neuronal differentiation, and activates the RhoA/Rho-associated kinase (ROCK)/c-jun N-terminal kinase (JNK) pathway. In addition, Wnt5a-induced neuronal differentiation was blocked by RhoA siRNA, as well as by a specific Rho-kinase inhibitor (Y27632) or a SAPK/JNK inhibitor (SP600125). Furthermore, treatment with RhoA siRNA, Y27632, or SP600125 suppressed the IL-1β-induced neuronal differentiation. Therefore, these results suggest that the sequential Wnt5a/RhoA/ROCK/JNK pathway is involved in IL-1β-induced neuronal differentiation of NPCs.

**Electronic supplementary material:**

The online version of this article (10.1186/s13041-018-0383-6) contains supplementary material, which is available to authorized users.

## Introduction

Interleukin-1 beta (IL-1β) is a pro-inflammatory cytokine, which is produced as an immune response to both injury and infection [1]. Increased production of IL-1β is involved in a variety of cellular activities, including cell proliferation, differentiation, and apoptosis [2]. IL-1β induces an intracellular signaling pathway, leading to altered expression of its target genes and the induction of a pro-inflammatory response [3]. It is considered that inflammatory processes stimulated by IL-1β are associated with an increased risk of neurodevelopmental disorders. Previous studies showed that IL-1β can aggravate the primary damage caused by infection of the central nervous system (CNS), and IL-1β deficient mice display reduced neuronal loss and infarct volumes after ischemic brain damage in in vivo studies [4, 5]. On the contrary, IL-1β showed beneficial effects on neuronal survival in cultures; it is highly expressed in the developing brain and can directly influence neural precursor cells (NPCs) [6]. In other in vitro models, IL-1β has been shown to stimulate the migration of cultured cortical neurons and promote neurite outgrowth [1, 7]. The findings in various neuronal culture models show that long-term exposure (3–5 days) to high concentrations of IL-1β (500 ng/ml) had neurotoxic effects; however, short-term exposure (1 day) to lower concentrations (10 ng/ml) did not [8]. Thus, it is important to elucidate whether IL-1β mediates neuronal differentiation to understand neural development and the pathogenesis of various neurodevelopmental disorders.

Wnt signaling plays an important role in embryogenesis and in the late stages of development, by regulating cell survival, growth, and, differentiation via various signaling pathways [9, 10]. Wnt signaling involves a group of pathways, such as the canonical Wnt/β-catenin pathway, the noncanonical Wnt/Ca^2+^ pathway, and the Wnt/planar cell polarity (PCP) pathway [11, 12]. Several Wnt isoforms (Wnt3b, Wnt5a, Wnt7a, and Wnt11) activate various signaling cascades during the maintenance of many organs and tissues [12–14]. A recent report suggested that Wnt5a plays a role in cell differentiation and specialization [15]. Wnt5a promotes the differentiation of mesenchymal stem cells (MSCs) into type II alveolar epithelial cells (AT II cells) [16] and acts as a repulsive guidance cue for cortical axons extending through the corpus callosum in vivo [17]. Wnt5a is also involved in IL-1β-mediated cell migration and differentiation. IL-1β-induced Wnt5a protein enhanced human corneal endothelial cell migration through the regulation of Cdc42 and RhoA [18], and silencing of Wnt5a prevents IL-1β-induced collagen type II degradation in rat chondrocytes [19]. Furthermore, Wnt5a signaling is involved in IL-1β-induced matrix metalloproteinase (MMP)-3-regulated proliferation of ES cell-derived odontoblast-like cells [20]. Although Wnt5a has been associated with the regulation of proliferation and differentiation in different cell types, little is known about the role of Wnt5a signaling in neuronal differentiation of NPCs.

Rho-family GTPases play an important role in regulating intracellular cytoskeletal and signaling pathways that facilitate axonal morphological changes [21, 22]. The most commonly studied Rho-GTPases are RhoA, Cdc42, and Rac1 [23]. Rho family GTPases serve as a molecular switch by converting from an inactive GDP-bound state to an active GTP-bound state and, once activated, they can interact with their specific effectors [24]. The GTP-bound form of RhoA causes Rho-associate kinase (ROCK) activation [25]. The RhoA/ROCK pathway is associated with various neuronal functions, such as migration, dendrite development, and axonal extension [25, 26]. The role of RhoA is complicated in axonal branching and growth cone formation [27]. In some cases, RhoA negatively regulates axon branch that inactivation of intracellular Rho to stimulate axon growth and regeneration [28]. In contrast, other studies indicated that RhoA is involved in the promotion of axon branching [29, 30]. It is, therefore, necessary to determine the distinct physiological functions of RhoA in neuronal differentiation of NPCs.

In the present study, we first investigated the role of IL-1β in neuronal differentiation of NPCs and found that IL-1β activated noncanonical Wnt5a signaling (Wnt5a/RhoA/ROCK/JNK pathway). We also demonstrated that IL-1β-induced noncanonical Wnt5a signaling is required for neuronal differentiation, especially for neurite outgrowth, suggesting that IL-1β can promote the neuronal differentiation of NPCs.

## Methods

### Materials

Coon’s modified Ham’s F-12 medium and human insulin were purchased from Sigma Chemical Co (St Louis, MO, USA). Penicillin/streptomycin solution, neurobasal medium, and B27 were purchased from Gibco (Grand Island, NY, USA), and bFGF, recombinant rat IL-1β, and recombinant Wnt5a were purchased from R&D Systems (Minneapolis, MN, USA). The following antibodies were purchased: anti NT3 (#SC-547) and anti Ngn1 (#SC-19231) from Santa Cruz Biotechnology (Santa Cruz, CA, USA); anti p-JNK (#9251S), anti JNK (#9252S), anti p-NF-κB (#3031S) and anti NF-κB (#6956S) from Cell Signaling Technology (Beverly, MA, USA); anti Wnt5a (#MABD136) from Merck (Darmstadt, Germany); anti β-tubulin type III (TUJ1) (#801202) from BioLegend (San Diego, CA, USA); anti calnexin (#ADI-SPA-860-F) from Enzo Life Sciences (Farmingdale, NY, USA). Fluorescein-(DTAF)-conjugated streptavidin (#016–010-084) was purchased from Jackson Immuno Research (Westgrove, PA, USA). Y27632 and SP600125 were purchased from Abcam (Cambridge, UK).

### Primary culture of neural precursor cells

Embryonic brain cortices from E14 rat embryos were mechanically triturated in Ca^2+^/Mg^2+^-free Hank’s balanced salt solution (Gibco), seeded at 2 × 10^5^ cells in 10-cm culture dishes (Corning Life Sciences, Acton, MA, USA), which were precoated with 15 μg/ml poly-L-ornithine (Sigma) and 1 μg/ml fibronectin (Invitrogen, Carlsbad, CA, USA). The cells were then cultured for 5–6 days in serum-free N2 medium supplemented with 20 ng/ml bFGF (R&D Systems Inc.). Cell clusters generated by precursor cell proliferation were dissociated in Hank’s balanced salt solution and plated at 2 × 10^4^ cells per well on coated 24-well plates, 2 × 10^5^ cells per well on coated 6-well plates, and 8 × 10^5^ cells per dish on coated 6-cm culture dishes. All experiments were performed using passage 1 (P1) neural precursor cells.

### Transient transfection of NSCs

*RhoA* (Dharmacon, Lafayette, CO, USA, Catalog No. L-095222-02), *Wnt5a* (Dharmacon, Catalog No. L-088203-02), *Control* (Dharmacon, Catalog No. D-001810-10) *or NF-κB p65* siRNA (Bioneer, Daejeon, KOREA, Catalog No. 1783704) were introduced into cells in knockdown experiments using a Nucleofector™ kit (Lonza, Basel, Switzerland, Catalog No. VPG-1003, Program A-031).

### Real time PCR and RT (reverse transcription)-PCR

cDNA was prepared using the total mRNA extracted from cells with TRIzol® reagent (Thermo Fisher Scientific); 2 μg samples of RNA were reverse-transcribed using random hexamer mixed primers. The cDNA thus formed was amplified by PCR using the following primers:

*Wnt3a* (5′-TGCAAATGCCACGGACTATC-3′ and 5′-AGACTCTCGGTGTTTCTCTACC-3′),

*Wnt5a* (5′-TGCCACTTGTATCAGGACCA-3′ and 5′-GGCTCATGGCATTTACCACT-3′),

*Wnt5b* (5′-CAAGCTGGAACTGACCAACA-3′ and 5′-AAAGCAACACCAGTGGAACC-3′),

*Wnt7a* (5′-CACAATTCCGAGAGCTAGGC-3′ and 5′-TAGCCTGAGGGGCTGTCTTA-3′),

*Wnt7b* (5′-GCTATCAGAAGCCGATGGAG-3′ and 5′-ACGTGTTGCACTTGACGAAG-3′),

*Nt3* (5′-GGCACACACACAGGAAGTGTC-3′ and 5′-CTGGACGTCAGGCACGGCCTGT-3′),

*Ngn1* (5′-ATGCCTGCCCCTTTGGAGAC-3′ and 5′-TGCATACGGTTGCGCTCGC-3′),

*p65 NF-κB* (5′-CTAGGAGGACTCGGGCTCTT-3′ and 5′-AGGAGCTCCACAGGACAGAA-3′),

*Gapdh* (5′-CTCGTCTCATAGACAAGATG-3′ and 5′-AGACTCCACGACATACTCAGCAC-3′).

The PCR conditions for amplification of *Wnt3a, Wnt5a, Wnt5b, Wnt7a, Wnt7b, Nt3, Ngn1, p65 NF-κB,* and *Gapdh* were as follows: denaturation at 94 °C for 30 s, annealing at 58 °C for 1 min, and extension at 72 °C for 1 min. 30 cycles were used for amplification of all cDNAs. The PCR products were analyzed on a 1.5% agarose gel. For real-time PCR, 5 μl of RT reaction product was amplified in duplicates at a final volume of 20 μl iQ™ SYBR®Green Super mix. Thermocycling conditions were 95 °C for 10 min, followed by 40 cycles of 95 °C for 15 s and 60 °C for 1 min. The primer sequences for real-time PCR were same as those used for RT-PCR, and all gene expression values were normalized to those of *Gapdh*.

### Western blot analysis

Cells were lysed in 20 mM Tris–HCl (pH 7.5) containing 150 mM NaCl, 1 mM Ethylenediaminetetraacetic acid (EDTA), 1 mM Ethylene glycol-bis(2-aminoethyl ether)-N,N,N′,N′-tetraacetic acid (EGTA), 2.5 mM sodium pyrophosphate, 1% Triton X-100, 1 mM phenylmethylsulfonyl fluoride (PMSF), and 1 mM Na_3_VO_4_. Protein samples (20–30 μg) were loaded on 10% SDS–polyacrylamide gels, electrophoresed, and transferred to nitrocellulose membranes (Amersham Pharmacia Biotech, Amersharm, UK) after electrophoresis. After blocking with 5% non-fat dried milk for 1 h, membranes were incubated with primary antibodies followed by incubation with HRP-conjugated secondary antibody (Anti-mouse IgG (#7076) and Anti-rabbit IgG (#7074)) (1:2000) (New England Biolabs, Beverly, MA, USA), and specific bands were detected by ECL (Amersham Pharmacia Biotech).

### RhoA activation assay

RhoA activity in cells was measured using Rho Activation assay kit (Merck, Catalog No. 17–294), according to the manufacturer’s instructions. Briefly, cells were washed with ice-cold PBS, and lysed with Mg^2+^ Lysis/Wash Buffer (MLB). Lysates were collected by centrifugation at 14,000×*g* for 5 min at 4 °C. Equal amounts of protein from cell lysates were incubated for 45 min at 4 °C with 20 μg of Rhotekin-RBD protein agarose beads. Pellets were washed with MLB and subjected to western blotting using the anti-RhoA antibody.

### Immunofluorescence staining

Cells were washed with PBS and fixed with 4% paraformaldehyde in PBS, followed by three washes with PBS at room temperature. They were then permeabilized with 0.1% Triton X-100 in PBS for 10 min, followed by three washes with PBS, and then blocked with 10% normal goat serum in PBS containing 0.5% Tween 20 for 1 h at room temperature. Next, the cells were incubated with the mouse monoclonal anti-β-tubulin type III (TUJ1) antibody (1:2000 dilution) primary antibody at 4 °C. Cells were then stained with streptavidin-conjugated secondary antibody (1:400) for 1 h before mounting with Vectashield (Vector Laboratories, Burlingame, CA, USA) containing 4, 6-diamidino-2-phenylindole (DAPI). Immunoreactive cells were detected using a TCS SP5 confocal imaging system (Leica Microsystems, Wetzlar, Germany) at magnification between 40× and 60 ×.

### Measurement of neurite outgrowth

Cells were cultured on coverslips coated with fibronectin in 24 well plates, fixed with 0.1% (*w*/*v*) picric acid/PBS containing 4% (w/v) paraformaldehyde, and incubated overnight at 4 °C with β-tubulin type III (TUJ1) antibody 1:2000. After incubation with Cy3-conjugated secondary antibody (Jackson Immuno Research Laboratories, PA, USA), cells were mounted on slides with Vectashield. TUJ1-positive cells were photographed using the TCS SP5 confocal imaging system (Leica Microsystems) and morphological characteristics were quantitated using Image J software (NIH-http//rsb.Info.nih.gov/ij/). The length of primary neurite was defined as the distance from the soma to the tip of the longest branch. For each graph, data on neurite length were generated from randomly selected areas of at least five independent cultures from three independent experiments and more than 100 cells were counted for each condition in each experiment.

### Statistical analysis

Cells were counted in randomly chosen (fractionator) microscope fields. Data are expressed as means ± SDs of three independent experiments. Differences were analyzed using the Student’s *t* test and were considered significant at *p* < 0.05.

## Results

### IL-1β-induced Wnt5a expression is required for neuronal differentiation

**Fig. 1:**
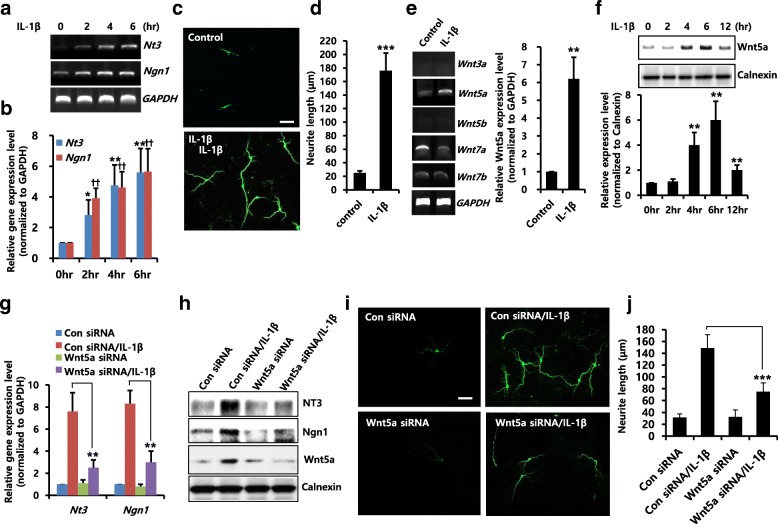
Effects of IL-1β-induced Wnt5a expression on neuronal differentiation. NPCs were treated with IL-1β (10 ng/ml) for the indicated time durations and mRNA levels of *Nt3*, *Ngn1* were analyzed by RT-PCR (**a**) and real-time RT-PCR (**b**). *n =* 3. Data are mean ± SD; Student’s *t* test. * *p* < 0.05, ** *p* < 0.01, ^††^*p* < 0.01 compared with 0 h control, for *Nt3* and *Ngn1* respectively. **c** and **d** NPCs were treated with IL-1β (10 ng/ml) for 3 days, and they were stained with anti-Tuj1 to visualize neurite extensions. Scale bar, 20 μm. **d** Neurite lengths were measured in randomly selected fields using three independent experiments. *n =* 3 per group. Data are mean ± SD; Student’s *t* test. *** *p* < 0.001 compared with untreated control. **e** NPCs were treated with IL-1β (10 ng/ml) for 2 h. mRNA levels of *Wnt3a, Wnt5a, Wnt5b, Wnt7a*, and *Wnt7b* were analyzed by RT-PCR (left). mRNA level of Wnt5a was analyzed by real-time RT-PCR (right). *n =* 3. Data are mean ± SD; Student’s *t* test. ** *p* < 0.01 compared with control. **f** NPCs were treated with IL-1β (10 ng/ml) for the indicated time durations, and cells were lysed. Western blotting was performed using anti-Wnt5a or anti-calnexin antibodies to detect the respective protein bands. Graphs show mean densities as percentage change for three independent experiments (*n =* 3). Band intensity was quantified with Quantity Ones® software. Data are mean ± SD; Student’s *t* test. ** *p* < 0.01 compared with 0 h control. **g** and **h** Cells were transiently transfected with control siRNA or Wnt5a siRNA for 48 h, and then treated for 6 h (**g**) or 2 days (**h**) with IL-1β (10 ng/ml). **g** mRNA levels of *Nt3* and *Ngn1* were analyzed by real-time RT-PCR. The results are based on three independent experiments (*n* = 3). Data are mean ± SD; Student’s *t* test. ** *p* < 0.01 compared with control siRNA/IL-1β. **h** Western blotting was performed using anti-NT3, anti-Ngn1, anti-Wnt5a or anti-calnexin antibodies to detect the respective protein bands (**i**). Cells were transiently transfected with control siRNA or Wnt5a siRNA for 48 h, and then treated for 3 days with IL-1β (10 ng/ml). They were then stained with anti-Tuj1. Scale bar, 20 μm. **j** Neurite lengths were measured in randomly selected fields using four independent experiments. *n* = 4 per group. Data are mean ± SD; Student’s *t* test. *** *p* < 0.001 compared with control siRNA/IL-1β

We first established the distribution of IL-1β in neuronal differentiation of NPCs. Nerve growth factors, such as NT3, NT4/5, and BDNF, together with the basic helix-loop-helix transcription factors neuro-D and neurogenin-1 (Ngn1) are closely associated with neuronal differentiation and can be used as markers of this process. Treatment with IL-1β increased the expression levels of NT3 and Ngn1 in a time-dependent manner (0–6 h) (Fig. 1a and b) and enhanced neurite outgrowth (Fig. 1c and d). Several studies suggest that Wnt signaling is involved in cell differentiation [10, 15, 16]; therefore, we examined the role of Wnt signaling in IL-1β-induced neuronal differentiation. As shown in Fig. 1e, stimulation with IL-1β increased mRNA level of Wnt5a, but not of other isoforms, and protein expression of Wnt5a induced by IL-1β gradually increased over 6 h and then decreased (Fig. 1f). We also showed that IL-1β-induced mRNA (Fig. 1g) and protein (Fig. 1h) expression levels of NT3 and Ngn1 were suppressed by Wnt5a knockdown using siRNA. In addition, IL-1β-induced neurite outgrowth was decreased because of knockdown of Wnt5a using siRNA (Fig. 1i and j), suggesting that IL-1β-mediated Wnt5a expression plays a role in neuronal differentiation of NPCs.

### Effect of NF-κB on IL-1β-induced Wnt5a expression and neurite outgrowth

**Fig. 2:**
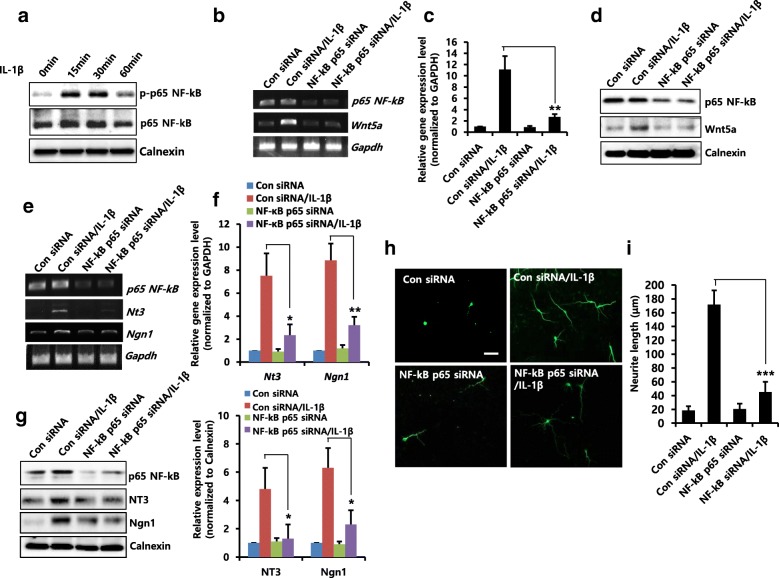
Effects of NF-κB on IL-1β–induced Wnt5a expression and neuronal differentiation. **a** NPCs were treated with IL-1β (10 ng/ml) for the indicated time durations, lysed, and harvested. Western blotting was performed using anti-p-p65 NF-κB, anti-p65 NF-κB, or anti-calnexin antibodies to detect the respective protein bands. **b**-**d** Cells were transiently transfected with control siRNA or NF-κB p65 siRNA for 48 h, and then treated for 2 h (**b** and **c**) or 6 h (**d**) with IL-1β (10 ng/ml). mRNA levels of *Wnt5a* were estimated by RT-PCR (**b**) and real-time RT-PCR (**c**). *n* = 3. Data are mean ± SD; Student’s *t* test. ** *p* < 0.01 compared with control siRNA/IL-1β. **d** Western blotting was performed using anti-p65 NF-κB, anti-Wnt5a, or anti-calnexin antibodies to detect the respective protein bands. **e** and **f** Cells were transiently transfected with control siRNA or NF-κB p65 siRNA for 48 h, and then treated for 6 h with IL-1β (10 ng/ml). mRNA levels of *Nt3* and *Ngn1* were analyzed by RT-PCR (**e**) and real-time RT-PCR (**f**). *n* = 3. Data are mean ± SD; Student’s *t* test. * *p* < 0.05, ** *p* < 0.01 compared with control siRNA/IL-1β. **g** Cells were transiently transfected with control siRNA or NF-κB p65 siRNA for 48 h, and then treated for 2 days with IL-1β (10 ng/ml). Western blotting was performed using anti-p65 NF-κB, anti-NT3, anti-Ngn1, or anti-calnexin antibodies to detect the respective protein bands. Graphs show mean densities as fold change for three independent experiments (*n* = 3). Band intensity was quantified with Quantity Ones® software. Data are mean ± SD; Student’s *t* test. *p* < 0.05 compared with control siRNA/IL-1β. **h** and **i** Cells were transiently transfected with control siRNA or NF-κB p65 siRNA for 48 h, and then treated for 3 days with IL-1β (10 ng/ml). They were then stained with anti-Tuj1. Scale bar, 20 μm. **i** Neurite lengths were measured in randomly selected fields using four independent experiments. *n* = 4 per group. Data are mean ± SD; Student’s *t* test. *** *p* < 0.001 as compared to control siRNA/IL-1β

The rat Wnt5a promoter contains both conserved and putative nuclear factor kappa B (NF-κB) binding sites [31] and its expression is NF-κB activity dependent [32]. To determine whether NF-κB affects IL-1β-induced Wnt5a expression, we first examined the phosphorylation of p65 NF-κB by IL-1β stimulation. IL-1β increased phosphorylation of NF-κB p65 within 30 min (Fig. 2a). We showed that IL-1β-induced mRNA (Fig. 2b and c) and protein (Fig. 2d) expression levels of Wnt5a were decreased due to knockdown of NF-κB p65 using siRNA, indicating that IL-1β-induced Wnt5a expression is NF-κB dependent. We further showed that IL-1β-induced mRNA (Fig. 2e and f) and protein (Fig. 2g) expression levels of NT3 and Ngn1 were inhibited by NF-κB p65 siRNA, and IL-1β-induced neurite outgrowth was decreased by NF-κB p65 knockdown (Fig. 2h and i). These results suggest that NF-κB activity is involved in IL-1β-induced Wnt5a expression and in the neurite outgrowth of NPCs.

### Wnt5a promotes neurite outgrowth through a RhoA/ROCK/JNK pathway

**Fig. 3:**
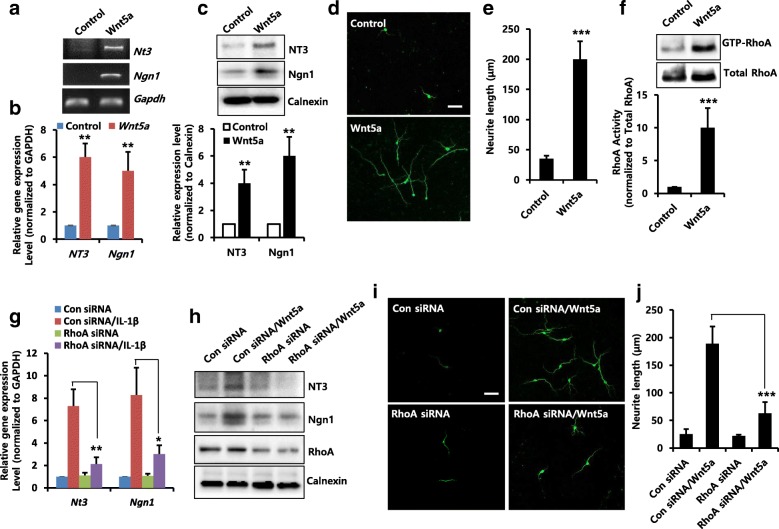
Effects of exogenous Wnt5a on Rho A activity and neuronal differentiation. NPCs were stimulated with Wnt5a (20 ng/ml) for 6 h, and mRNA levels of *Nt3* and *Ngn1* were analyzed by RT-PCR (**a**) and real time-RT-PCR (**b**). *n* = 3. Data are mean ± SD; Student’s *t* test. ** *p* < 0.01 compared with the untreated control. **c** Cells were treated with Wnt5a (20 ng/ml) for 2 days, and then lysed and harvested. Western blotting was performed using anti-NT3, anti-Ngn1, or anti-calnexin to detect respective protein bands. Graphs show mean densities as fold change for three independent experiments (*n* = 3). Band intensity was quantified with Quantity Ones® software. Data are mean ± SD; Student’s *t* test. ** *p* < 0.01 compared with untreated cells. **d** and **e** NPCs were treated with Wnt5a (20 ng/ml) for 3 days, and were stained with anti-Tuj1 to visualize neurite extensions. Scale bar, 20 μm. **e** Neurite lengths were measured in randomly selected fields using three independent experiments. *n* = 3 per group. Data are mean ± SD; Student’s *t* test. ** *p* < 0.01 compared with untreated control. **f** GTP-loaded RhoA activity was measured using a pull-down assay, as described in the Materials and Methods section, after treatment of cells for 15 min with Wnt5a (20 ng/ml). The data were normalized to the amount of total RhoA. Graphs show mean densities as fold change for four independent experiments (*n* = 4). Band intensity was quantified with Quantity Ones® software. Data are mean ± SD; Student’s *t* test. *** *p* < 0.001 compared with the untreated control. **g** and **h** Cells were transiently transfected with control siRNA or RhoA siRNA for 48 h, and then treated for 6 h (**g**) or 2 days (**h**) with Wnt5a (20 ng/ml). **g** mRNA levels of *Nt3* and *Ngn1* were analyzed by real-time RT-PCR. *n* = 3. Data are mean ± SD; Student’s *t* test. * *p* < 0.05, ** *p* < 0.01 compared with control siRNA/Wnt5a. **h** Western blotting was performed using anti-NT3, anti-Ngn1, anti-RhoA, or anti-calnexin to detect the respective protein bands. **i** and **j** Cells were transiently transfected with control siRNA or RhoA siRNA for 48 h, and then treated for 3 days with Wnt5a (20 ng/ml). They were then stained with anti-Tuj1. Scale bar, 20 μm. **j** Neurite lengths were measured in randomly selected fields using five independent experiments. *n* = 5 per group. Data are mean ± SD; Student’s *t* test. *** *p* < 0.001 compared with control siRNA/Wnt5a

We tested whether exogenous Wnt5a could enhance neuronal differentiation. As shown in Fig. 3a-c, mRNA (Fig. 3a and b) and protein (Fig. 3c) levels of NT3 and Ngn1 were increased by Wnt5a treatment. Exogenous Wnt5a also markedly increased neurite outgrowth (Fig. 3d and e), indicating the importance of Wnt5a in neuronal differentiation of NPCs. Previous studies suggest that RhoA plays a central role in dendritic development, and that differential activation of Rho-related GTPases contributes to the generation of morphological diversity in the developing cortex [24]. As Wnt5a has recently been found to be involved in the regulation of RhoA [18], we examined the effect of Wnt5a on the activity of RhoA. Treatment with Wnt5a increased RhoA activity (Fig. 3f). Next, we examined the role of RhoA in Wnt5a-induced neuronal differentiation. As shown in Fig. 3g and h, Wnt5a-induced mRNA and Protein levels of NT3 and Ngn1 were decreased by RhoA knockdown with siRNA. Moreover, Wnt5a-induced neurite outgrowth was inhibited by RhoA knockdown (Fig. 3i and j). We further showed that the ROCK inhibitor Y27632 suppressed Wnt5a-induced increase in mRNA (Additional file 1: Figure S1A and B) and protein levels of NT3 and Ngn1 (Additional file 1: Figure S1C), and also Y27632 inhibited neurite outgrowth induced by Wnt5a (Additional file 1: Figure S1D and E), indicating that RhoA/ROCK pathway is implicated in Wnt5a-induced neuronal differentiation.

**Fig. 4:**
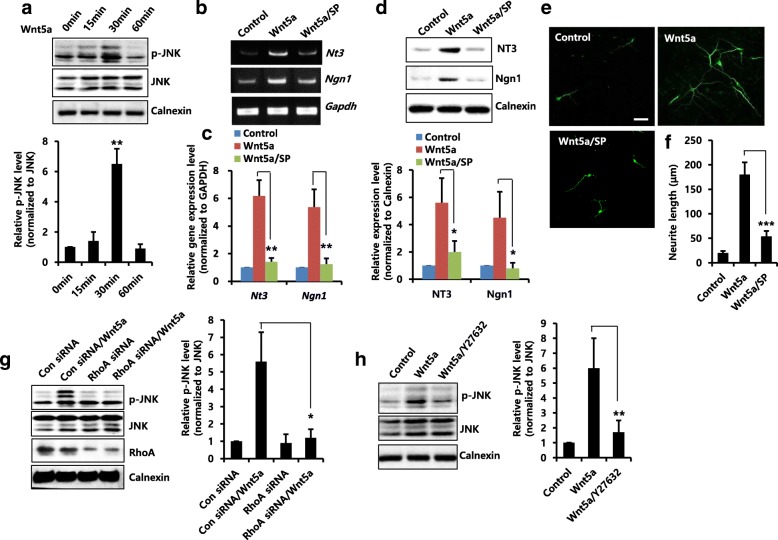
Effects of Wnt5a/RhoA/ROCK pathway on JNK activation and neuronal differentiation. **a** NPCs were treated with Wnt5a (20 ng/ml) for the indicated time duration, lysed and harvested. Western blotting was performed using anti-p-JNK, anti-JNK, or anti-calnexin to detect the respective protein bands. Graphs show mean densities as fold change for three independent experiments (*n* = 3). Band intensity was quantified with Quantity Ones® software. Data are mean ± SD; Student’s *t* test. ** *p* < 0.01 compared with 0 min. **b** and **c** Cells were pretreated with 2 μM SP600125 (SP) for 1 h and stimulated with Wnt5a (20 ng/ml) for 6 h. mRNA levels of NT3 and Ngn1 were analyzed by RT-PCR (**b**) and real-time RT-PCR (**c**). *n* = 3. Data are mean ± SD; Student’s *t* test. ** *p* < 0.01 as compared to Wnt5a-treated cells. **d** Cells were pretreated with 2 μM SP for 1 h and treated with Wnt5a (20 ng/ml) for 2 days. Western blotting was performed using an anti-NT3, anti-Ngn1, or anti-calnexin to detect the respective protein bands. Graphs show mean densities as fold change from three independent experiments (*n* = 3). Band intensity was quantified with Quantity Ones® software. Data are mean ± SD; Student’s *t* test. * *p* < 0.05 compared with Wnt5a-treated cells. **e** and **f** Cells were pretreated with 2 μM SP for 1 h and treated with Wnt5a (20 ng/ml) for 3 days. They were then stained with anti-Tuj1. Scale bar, 20 μm. **f** Neurite lengths were measured in randomly selected fields using four independent experiments. *n* = 4 per group. Data are mean ± SD; Student’s *t* test. *** *p* < 0.001 compared with Wnt5a-treated cells. **g** Cells were transiently transfected with control siRNA or RhoA siRNA for 48 h, and then treated for 30 min with Wnt5a (20 ng/ml). Western blotting was performed using anti-p-JNK, anti-JNK, anti-RhoA, or anti-calnexin to detect the respective protein bands. Graphs show mean densities as fold change from three independent experiments (*n* = 3). Band intensity was quantified with Quantity Ones® software. Data are mean ± SD; Student’s *t* test. * *p* < 0.05 as compared to control siRNA/Wnt5a. **h** Cells were pretreated with 5 μM Y27632 for 1 h and stimulated with Wnt5a (20 ng/ml) for 30 min. Western blotting was performed using anti-p-JNK, anti-JNK, or anti-calnexin to detect the respective protein bands. Graphs show mean densities as fold change from three independent experiments (*n* = 3). Band intensity was quantified with Quantity Ones® software. Data are mean ± SD; Student’s *t* test. ** *p* < 0.01 compared with Wnt5a-treated cells

The JNK (c-Jun N-terminal Kinase), also known as stress-activated protein kinase, has extensive implications in understanding important biological processes, such as cell growth, differentiation, tissue development and regeneration [33, 34]. Previous studies reported that JNK mediates neural differentiation, induction of the neural-specific gene neurofilament light chain (NFLC) and development of embryonic stem cells [35, 36]. Interestingly, Wnt5a/JNK signaling contributed to the differentiation of mesenchymal stem cells [16]. Thus, we examined whether JNK activation is involved in Wnt5a-induced neuronal differentiation of NPCs. As shown in Fig. 4a, Wnt5a treatment increased phosphorylation of JNK. When the cells were pretreated with a JNK inhibitor, SP600125, Wnt5a-induced mRNA (Fig. 4b and c) and protein (Fig. 4d) levels of NT3 and Ngn1 were decreased. Moreover, Wnt5a-induced neurite outgrowth was significantly decreased by SP600125, indicating that JNK activation is related to Wnt5a-induced neuronal differentiation (Fig. 4e and f). Next, we determined the involvement of the RhoA/ROCK pathway on Wnt5a-mediated JNK activation. As shown in Fig. 4g, Wnt5a-induced phosphorylation of JNK was decreased by RhoA siRNA. Furthermore, pre-treatment with Y27632 reduced Wnt5a-induced phosphorylation of JNK (Fig. 4h). Therefore, these results suggest that Wnt5a-induced neuronal differentiation is regulated by the RhoA/ROCK/JNK pathway.

### Evaluation of Wnt5a-mediated pathway on IL-1β-induced neuronal differentiation

**Fig. 5:**
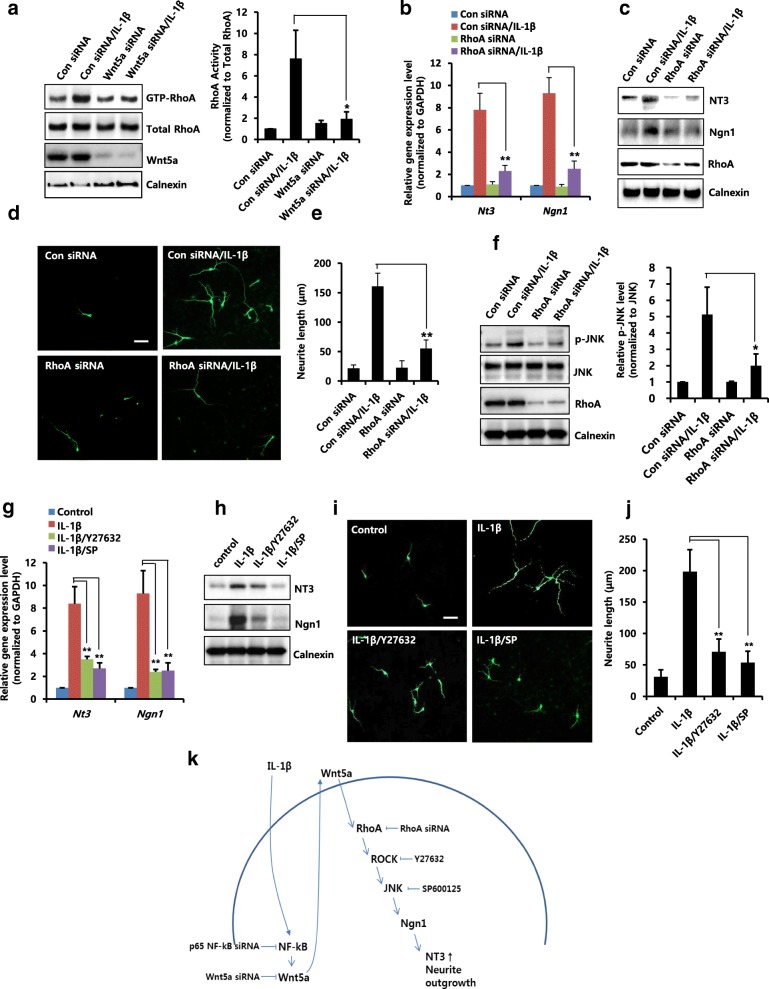
Effects of IL-1β on Wnt5a-mediated signaling and neuronal differentiation. **a** NPCs were transiently transfected with control siRNA or Wnt5a siRNA for 48 h, and then incubated for 15 min with IL-1β (10 ng/ml). GTP-loaded RhoA activity was measured using a pull-down assay, as described in the Materials and Methods section. The data were normalized to the amount of total RhoA. Graphs show mean densities as fold change from three independent experiments (*n* = 3). Band intensity was quantified with Quantity Ones® software. Data are mean ± SD; Student’s *t* test. * *p* < 0.05 compared with control siRNA/IL-1β. **b** and **c** Cells were transiently transfected with control siRNA or RhoA siRNA for 48 h, and then incubated for 6 h (B) or 2 days (**c**) with IL-1β (10 ng/ml). **b** mRNA levels of *Nt3* and *Ngn1* were analyzed by real-time RT-PCR. *n* = 3. Data are mean ± SD; Student’s *t* test. ** *p* < 0.01 as compared to control siRNA/IL-1β. **c** Cells were transiently transfected with control siRNA or RhoA siRNA for 48 h, and then treated for 2 days with IL-1β (10 ng/ml). Western blotting was performed using anti-NT3, anti-Ngn1, anti-RhoA, or anti-calnexin antibodies to detect the respective protein bands. **d** and **e** Cells were transiently transfected with control siRNA or RhoA siRNA for 48 h, and then incubated for 3 days with IL-1β (10 ng/ml). They were then stained with anti-Tuj1. Scale bar, 20 μm. **e** Neurite lengths were measured in randomly selected fields using three independent experiments. *n* = 3 per group. Data are mean ± SD; Student’s *t* test. ** *p* < 0.01 compared with control siRNA/IL-1β. **f** Cells were transiently transfected with control siRNA or RhoA siRNA for 48 h, and then treated for 30 min with IL-1β (10 ng/ml). Western blotting was performed using anti-p-JNK, anti-JNK, anti-RhoA or anti-calnexin antibodies to detect the respective protein bands. Graphs show mean densities as fold change for three independent experiments (*n* = 3). Band intensity was quantified with Quantity Ones® software. Data are mean ± SD; Student’s *t* test. * *p* < 0.05 compared with control siRNA/IL-1β. **g** and **h** Cells were pretreated with 5 μM Y27632 or 2 μM SP for 1 h, and then treated with IL-1β (10 ng/ml) for 6 h (**g**) or 2 days (**h**). **g** mRNA levels of *Nt3* and *Ngn1* were analyzed by real-time RT-PCR. *n* = 3. Data are mean ± SD; Student’s *t* test. ** *p* < 0.01 compared with IL-1β-treated cells. **h** Cells were pretreated with 5 μM Y27632 or 2 μM SP for 1 h, and then treated with IL-1β (10 ng/ml) for 2 days. Western blotting was performed using anti-NT3, anti-Ngn1 or anti-calnexin antibodies to detect the respective protein bands. (I and J) Cells were pretreated with 5 μM Y27632 or 2 μM SP for 1 h, and then treated with IL-1β (10 ng/ml) for 3 days. They were then stained with anti-Tuj1. Scale bar, 20 μm. **j** Neurite lengths were measured in randomly selected fields using three independent experiments. *n* = 3 per group. Data are mean ± SD; Student’s *t* test. ** *p* < 0.01 as compared to IL-1β-treated cells. **k** Proposed model for the signaling pathway in IL-1β-mediated neurite outgrowth of cortical NPCs. The model suggests that IL-1β-induced Wnt5a plays a major stimulatory role in neuronal differentiation and that it acts through the RhoA/ROCK/JNK pathway, leading to neurite outgrowth

We next examined whether the Wnt5a-mediated RhoA/ROCK/JNK pathway is involved in IL-1β-induced neuronal differentiation. We showed that IL-1β-induced RhoA activity was significantly decreased due to knockdown of Wnt5a using siRNA (Fig. 5a). We also found that IL-1β-induced mRNA (Fig. 5b) and protein (Fig. 5c) levels of NT3 and Ngn1 were decreased by RhoA siRNA, and IL-1β-induced neurite outgrowth was significantly inhibited by RhoA siRNA (Fig. 5d and e). In addition, RhoA knockdown with siRNA decreased IL-1β-induced phosphorylation of JNK (Fig. 5f). Furthermore, pre-treatment with Y27632 or SP600125 inhibitors not only suppressed IL-1β-induced mRNA and protein levels of NT3 and Ngn1 (Fig. 5g and h) but also decreased IL-1β-induced neurite outgrowth (Fig. 5i and j). These results suggest that Wnt5a/RhoA/ROCK/JNK pathway is involved in IL-1β-induced neuronal differentiation of NPCs.

## Discussion

IL-1β is known to be a key modulator of stress and inflammation in the CNS. Neuroinflammation is implicated in the pathophysiology of many psychiatric and neurodegenerative disorders where cognitive dysfunction and reductions in neurogenesis are evident, including Alzheimer’s disease (AD), Parkinson’s disease (PD), and depression [2]. IL-1β has been shown to negatively influence the proliferation and differentiation of hippocampal NPCs [37], and that it impairs neurotrophin-induced neuronal cell survival [38, 39]. In the spinal cord, IL-1β has been implicated in extensive inflammation and progressive neuro-degeneration after ischemic and traumatic injury [40, 41]. These results indicate that IL-1β might be involved in the pathogenesis of some neurodevelopmental disorders. However, there is increasing evidence that inflammation-associated cytokines can play a key role in stimulating neurite outgrowth and regeneration [42, 43]. Recent studies have provided an indication that IL-1β is able to stimulate the migration of cultured cortical neurons [7], and the potent neurotropic action of IL-1β leads to rapid neurite growth [44]. In this study, we found that stimulation with IL-1β facilitates neurite outgrowth, as well as increases the expression levels of neuronal factors, such as NT3 and Ngn1. Moreover, IL-1β-induced Wnt5a expression has a critical role in neuronal differentiation of NPCs. These results demonstrate a novel physiological function of IL-1β in neuronal differentiation of cortical NPCs.

Recently, Wnt5a, identified as an axon guidance cue, was shown to activate a non-canonical pathway essential for cortical axonal morphogenesis. In this study, we found that the Wnt5a/RhoA/ROCK/JNK pathway is required for IL-1β-mediated neurite outgrowth of NPCs. The effect of Wnt5a signaling on axon outgrowth of cortical neurons is consistent with a recent study showing that Wnt5a increases axon length and promotes axon differentiation of dissociated hippocampal neurons [45]. In addition, a recent study suggests that Wnt5a promotes axonal growth through the regulation of calcium signaling during neuronal polarization [46]. In contrast, a previous study has shown that Wnt5a acts via Ryk receptors to inhibit the neurite outgrowth of sensorimotor cortex [47]. The different roles of Wnt5a in neurons are probably related to their activity dependent mechanisms. Therefore, it is necessary to elucidate a more detailed mechanism of Wnt5a signaling in developing neurite outgrowth and axon guidance.

Axon outgrowth and navigation during synapse formation are regulated by extracellular signals. The cytoskeletal mechanisms of axonal branching are regulated by Rho GTPases [23]. Recent reports suggest that the Rho GTPases, including RhoA, Rac1, and Cdc42 play a central role in dendritic development, and that differential activation of Rho-related GTPases contributes to the generation of morphological diversity in the developing cortex [24, 48]. Cdc42 and Rac1 facilitate axonal branching and growth cone formation [21]. However, RhoA activity in axonal branch regulation is complex. It has been shown that RhoA negatively regulated axon formation [26]. RhoA/ROCK suppresses axonal protrusive activity by negatively regulating the filopodia from actin patches [27]. In contrast, our present study showed that RhoA/ROCK signaling induced by IL-1β or Wnt5a promotes neurite outgrowth, as well as increases the expression levels of neuronal factors, such as NT3 and Ngn1. In addition, recent studies reported that RhoA/ROCK pathway increased neurite outgrowth in hippocampal immortalized cells [49], and inhibition of RhoA activity reduces axonal branching in hippocampal neurons of embryonic mice [29]. These findings are consistent with our study showing that RhoA/ROCK signaling plays a positive role in neurite outgrowth. Considering that RhoA plays a different role in the regulation of neurite outgrowth depending on the cell environment (culture conditions and type of stimulation) or cell types, it will be necessary to determine the specific contribution of RhoA activity to all components of cytoskeletal mechanics and neurite outgrowth.

Here, we demonstrate a novel function of IL-1β in neuronal differentiation of NPCs. The findings of the present study are summarized in Fig. 5k. First, we found that stimulation with IL-1β promotes neuronal differentiation. Second, IL-1β induces Wnt5a expression through NF-κB activity. Third, IL-1β-induced Wnt5a is required for neuronal differentiation through a RhoA/ROCK/JNK pathway. Taken together, we conclude that IL-1β promotes neuronal differentiation through a Wnt5a/RhoA/ROCK/JNK pathway in cortical NPCs.

## Additional file

Additional file 1: **Figure S1.** NPCs were pretreated with 5 μM Y27632 for 1 h and stimulated with Wnt5a (20 ng/ml) for 6 h. mRNA levels of Nt3 and Ngn1 were analyzed by RT-PCR (A) and real time-RT-PCR (B). *n* = 3. Data are mean ± SD; Student’s *t* test. * *p* < 0.05 compared with Wnt5a-treated cells. (C) Cells were pretreated with 5 μM Y27632 for 1 h and stimulated with Wnt5a (20 ng/ml) for 2 days. Western blotting was performed using an anti-NT3, anti-Ngn1, or anti-calnexin antibodies to detect the respective protein bands. (D and E) Cells were pretreated with 5 μM Y27632 for 1 h and stimulated with Wnt5a (20 ng/ml) for 3 days. They were stained with anti-Tuj1. Scale bar, 20 μm. (E) Neurite lengths were measured in randomly selected fields using three independent experiments. *n* = 3 per group. Data are mean ± SD; Student’s *t* test. ** *p* < 0.01 compared with Wnt5a-treated cells. (PPTX 419 kb)

## Abbreviations

CNS: Central nervous system
IL-1β: Interleukin-1 beta
JNK: c-jun N-terminal kinase
NF-κB: Nuclear factor kappa B
Ngn1: Neurogenin1
NPCs: Neural precursor cells
NT3: Neurotrophin-3
ROCK: Rho-associated kinase
