# Supplementary figures and images for: Interleukin-1 beta promotes neuronal differentiation through the Wnt5a/RhoA/JNK pathway in cortical neural precursor cells
Source: Mol Brain. 2018 Jul 4;11:39. doi: 10.1186/s13041-018-0383-6 (PMC6033214; doi:10.1186/s13041-018-0383-6)

## Slide 1
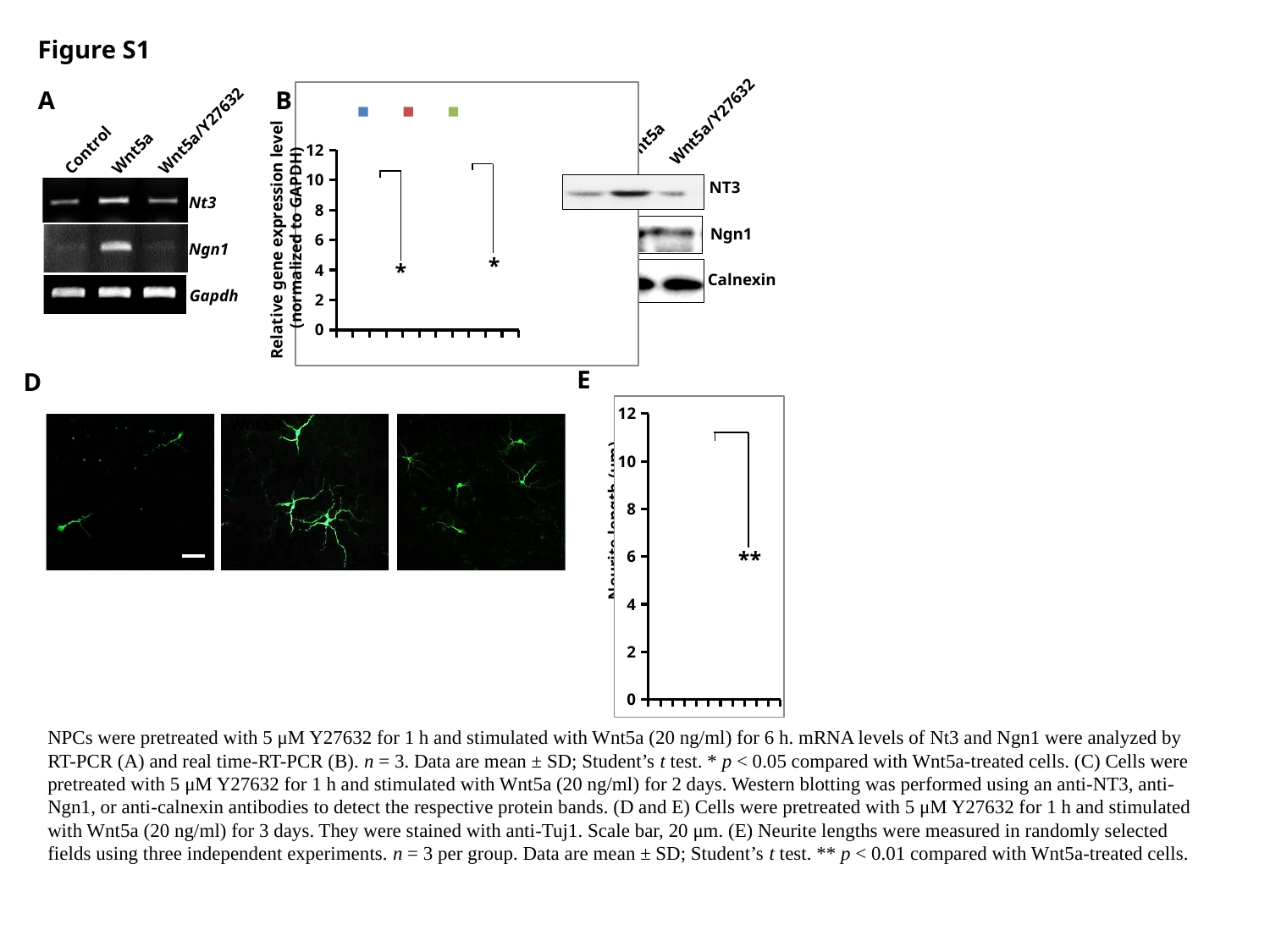

Supplement: Supplementary file 1 — Figure S1. NPCs were pretreated with 5 μM Y27632 for 1 h and stimulated with Wnt5a (20 ng/ml) for 6 h. mRNA levels of Nt3 and Ngn1 were analyzed by RT-PCR (A) and real time-RT-PCR (B). n = 3. Data are mean ± SD; Student’s t test. * p < 0.05 compared with Wnt5a-treated cells. (C) Cells were pretreated with 5 μM Y27632 for 1 h and stimulated with Wnt5a (20 ng/ml) for 2 days. Western blotting was performed using an anti-NT3, anti-Ngn1, or anti-calnexin antibodies to detect the respective protein bands. (D and E) Cells were pretreated with 5 μM Y27632 for 1 h and stimulated with Wnt5a (20 ng/ml) for 3 days. They were stained with anti-Tuj1. Scale bar, 20 μm. (E) Neurite lengths were measured in randomly selected fields using three independent experiments. n = 3 per group. Data are mean ± SD; Student’s t test. ** p < 0.01 compared with Wnt5a-treated cells. (PPTX 419 kb) [file 13041_2018_383_MOESM1_ESM.pptx]
